# Novel Optical Design of Large Aperture Supported Standard Wide Zoom Lens

**DOI:** 10.3390/s25164927

**Published:** 2025-08-09

**Authors:** Youngmin Na, Jaemyung Ryu, Hojong Choi

**Affiliations:** 1 Department of Optical Engineering, Kumoh National Institute of Technology, 350-27 Gumi-daero, Gumi 39253, Republic of Korea; dudals0510@kumoh.ac.kr; 2 Department of Electronic Engineering, Gachon University, 1342 Seongnam-daero, Sujeong-gu, Seongnam 13120, Republic of Korea

**Keywords:** standard wide zoom lens, large aperture, optical design

## Abstract

In this study, we designed a high-speed 16–50 mm F/1.8–2.8 zoom lens for advanced photo system type-C image sensors. Notably, this is the first attempt to implement a bright aperture of F/1.8 at the wide-angle end of a zoom lens. Compared to existing designs with fixed apertures of F/2.0–2.8 or F/2.8, our lens achieves a brighter open aperture while effectively suppressing aberrations and maintaining high resolution. Spherical aberration, chromatic aberration, and distortion are effectively controlled using three aspherical lenses and one extra-low dispersion lens. As a result, a modulation transfer function performance of over 80% at the center is achieved, even at F/1.8 in the wide-angle range. To enhance focusing performance, we adopted a second lens group with low aberration sensitivity as the autofocus driving group, contributing to reduced mechanical weight and improved stability. This design successfully realizes a high-speed zoom lens that delivers resolution comparable to or better than existing optical systems with brighter apertures, while also maintaining a well-balanced trade-off between optical performance and product size.

## 1. Introduction

Conventional standard zoom lens designs for advanced photo system type-C (APS-C) image sensors have primarily focused on achieving a balance between optical performance and design constraints within F/2.0–2.8 variable apertures or a constant F/2.8 aperture range [1]. While such designs offer stable aberration control and sufficient resolution based on aperture settings, they fall short of meeting the demands of modern imaging environments, which increasingly require brighter apertures, particularly in the wide-angle range.

The need for shallow depth of field to achieve background blur in portraiture and adequate exposure for bright photos in dark indoor scenarios has led to a growing demand for zoom lenses with brighter apertures [2,3]. Consequently, the development of a high-speed zoom lens with an aperture as bright as F/1.8 has become increasingly necessary. However, achieving an F/1.8 aperture at wide-angle focal lengths presents significant challenges, including the need for high optical resolution, precise aberration correction, weight reduction, and overcoming mechanical design constraints [4].

There have been limited studies over the past decade on zoom lens designs supporting large apertures. Optical systems with low f-numbers generally require large apertures, which complicates aberration correction and mechanical configuration [5]. Recent research has explored various methods to suppress aberrations in such systems. Yamanaka proposed a design that effectively mitigates aberrations in large-diameter lenses, achieving high resolution while maintaining bright apertures [6]. Additionally, minimizing lens group weight is essential due to the torque limitations of autofocus (AF) motors [7]. Lens group weight directly affects the performance, speed, and precision of AF systems [8].

To ensure stable aberration performance at low f-numbers, some studies have designated the second lens group—characterized by low aberration sensitivity—as the AF driving group. This approach minimizes variation in the spacing between the first and second groups and controls the optical path of peripheral rays after the second group [9]. Zhang proposed a zoom optical system structured with four lens groups and focused on design optimization for miniaturization, weight reduction, and zoom stability based on a stable imaging principle [10]. Ikeda introduced a zoom system that combines a wide-angle and compact design by using a positively powered (convex) first lens group instead of a negatively powered (concave) one. This configuration achieved a fixed F/2.8 aperture and improved aberration control over a focal length range of 18–50 mm [11].

In contrast, our design achieves similar levels of wide-angle coverage, miniaturization, and aberration suppression while delivering a brighter F/1.8 aperture. Furthermore, we propose a differentiated high-speed zoom design with an expanded angle of view starting from a wider 16 mm and maintaining an F/1.8 aperture. Shibata et al. proposed using a rear lens group near the image plane as the focusing unit to reduce the weight of the moving lens group, enabling faster AF by fixing the large-diameter front elements [12]. Our approach differs by assigning the second lens group (G2), with low aberration sensitivity, as the focusing unit. We designed the system to be compatible with small stepping motors (STM), reducing the focusing group weight to under 20 g—achieving performance comparable to or exceeding rear-focusing systems in both weight and aberration control. Nakayama achieved a large diameter and simultaneous miniaturization [13]. By separating the second lens group (G2) and third lens group (G3) and setting only G3 as the focusing lens group, the weight and speed of the focus lens group were reduced. However, there is a problem in that the complexity of optical system control and mechanical design increases because this configuration requires a total of six lens groups to move. Because the proposed product is based on the premise of aberration suppression under the condition of a fixed aperture of F/2.8, there is a clear difference in technical difficulty compared with our design, which achieves the same level of aberration suppression and resolution while maintaining a bright aperture value of F/1.8 in the wide-angle range. In other words, our proposed idea achieved both optical performance and mechanical efficiency by reducing the number of moving groups and the weight of the focus lens group under design conditions with a wider open aperture range. Meanwhile, Heo proposed a technical approach for miniaturizing and reducing the weight of the AF lens group to implement a bright aperture [14]. However, the proposed method showed limitations in effectively suppressing aberrations and maintaining overall system performance. Specifications and performance comparisons with existing systems are discussed later. Due to the technical challenges outlined above, there are currently no commercially available zoom lenses offering an F/1.8 aperture at wide angles. Achieving high resolution and effective aberration control at such apertures remains extremely difficult. Moreover, realizing a high-speed zoom lens that simultaneously delivers high resolution, compactness, and low weight—without increasing the number of elements or aspherical surfaces—is especially challenging.

In this study, we designed a high-speed standard zoom lens with a focal length range of 16–50 mm and an F/1.8–2.8 aperture for APS-C image sensors [15]. This design represents a significant technical breakthrough as the first attempt to overcome current limitations by achieving an F/1.8 aperture, especially at wide-angle settings. By minimizing the total number of lens elements and aspherical surfaces, we effectively suppressed major aberrations—including spherical aberration, chromatic aberration, field curvature, and distortion—using three aspherical lenses and one extra-low dispersion (ED) lens.

Modulation transfer function (MTF) analysis confirmed that high-resolution performance was maintained across both wide-angle and telephoto ranges, with central resolution exceeding 80% at 30 lp/mm under wide-angle F/1.8 conditions. Furthermore, by using a second lens group with low aberration sensitivity as the AF drive unit, the system allows for lightweight motor operation and maintains stable aberration characteristics during focusing.

Overall, the proposed design achieves equal or superior resolution at a brighter maximum aperture compared to current F/2.0–2.8 or constant F/2.8 zoom lenses, while satisfying the combined demands of optical performance and mechanical efficiency. As such, it offers strong potential as a practical solution for developing high-performance mirrorless camera lenses.

The rest of the paper is organized as follows. Section 2 compares our design with existing optical systems and describes the system’s mechanism and optical path. Section 3 presents the obtained MTF and aberration characteristics. Section 4 provides a summary of the proposed approach.

## 2. Materials and Methods

The demand for crop-body mirrorless cameras using APS-C sensors is increasing; consequently, the demand for suitable high-performance lenses is also growing. However, the lens range for APS-C sensors is still quite narrow [16]. Most of the lenses developed to date with a focal length of 16–50 mm have maximum apertures of F/2.8 or smaller, with the highest-performing lenses offering F/2.0–2.8 apertures. Therefore, in this study, we designed a high-performance standard wide-angle zoom optical system with a brighter aperture of F/1.8–2.8. Specifically, the f-number was reduced to 1.8 at the wide-angle end, allowing sufficient light intake and shutter speed, even in dark environments.

Zoom lenses are much more difficult to design with high optical performance and low f-numbers compared to prime lenses, which have a fixed focal length, because they must maintain consistent image quality across a wide focal range [17,18,19]. Most optical lenses developed to date in the 16–50 mm range are F/2.0–2.8 lenses. Therefore, we designed a high-performance standard wide-angle zoom optical system with a brighter aperture of F/1.8–2.8.

In Table 1, different standard wide-angle zoom lens designs and key specifications of the optical systems are compared. Table 1 presents lenses with the same focal length range (16 to 50 mm) in different optical layouts. The first optical system used 17 lenses—one more than the third optical system (Table 1c) and one fewer than the second system (Table 1b)—and also included three aspherical lenses. The minimum focusing distance (MOD), which refers to the distance from the subject, was set to 0.3 m in our design, similar to other optical systems.

Table 1a lists the specifications of the designed optical system, while Table 1b,c are organized based on commercial high-performance zoom lens products with the same focal length. After reflecting on the design structure of the actual product described in published patents, an optical layout was developed [13,14]. The design used in this study was a standard wide-angle zoom lens for APS-C sensors with an aperture value of F/1.8–2.8, which is brighter than the previous highest specification of F/2.0–2.8.

This was achieved by effectively controlling aberrations without adding extra lenses or internal aspherical lenses, rather than simply lowering the f-number. Therefore, through design optimization and a group deployment strategy, stable imaging can be achieved, even in high-speed shutter and low-light environments, without degradation of optical performance.

In particular, implementing F/1.8 under existing standards typically requires large-diameter lenses and an increased number of lens groups. However, this design achieves high performance with a limited number of lenses (17 in total), including three aspherical lenses and one extra-low dispersion (ED) lens. These results suggest new possibilities for implementing high-aperture zoom lens designs for crop-sensor-based mirrorless systems. Therefore, this study can serve as an important technical reference for future high-performance lens development.

The design of this study is a retro-focus type, where the front group has negative power based on the aperture, and the back group has positive power to diverge light and then converge it [20]. It consists of a total of 17 lenses and uses three aspherical lenses and one ED lens, so that there is no significant performance degradation due to increased aberrations, even at the F/1.8 specification. A total of four groups move to adjust the focus; all groups except Group 2 move toward the subject, and Group 2, which is the focus group, moves toward the image plane to adjust the focus point.

We designed the focal length to change naturally from wide-angle (16 mm) to telephoto (50 mm), while the aperture changes from F/1.8 to F/2.8, accordingly.

The optical system was designed using CODE V optical design software (Ver. 2023.03, Synopsys Inc., Sunnyvale, CA, USA). This study focuses on a zoom lens for an APS-C sensor, which typically has a sensor size of about two-thirds that of a full-frame sensor. In general, optical system design involves optimizing target specifications based on previously developed system data. However, due to the limited design data for zoom lenses with a 16–50 mm focal length for APS-C sensors, we based our design on a standard zoom lens with a 24–70 mm focal length for full-frame cameras, where more design data are available. This was scaled down by approximately two-thirds to fit the APS-C sensor, resulting in a focal length of about 16–46.7 mm—close to the target 16–50 mm range. Thus, we adjusted the design to suit the APS-C sensor.

A zoom lens is an optical system in which the effective focal length (EFL) or magnification changes continuously, while the position of the image plane remains fixed [20]. In such systems, focal length variation or magnification requires the movement of specific lens groups [21]. As the distances between lens groups change continuously, the angle of view also changes [22].

The state in which the angle of view changes continuously through space variation between each group is called zooming [23]. The relationship between the imaging element and the focal length can be expressed as Equation (1) [24]:

(1) y=ftanθ

When the size of the opposite angle of the image sensor is *y*, the focal length is *f*, and the angle of view is *θ*, the size of the image sensor is always the same as the camera sensor. Therefore, the relationship between angle of view and focal length can be expressed as follows in Equation (2).

(2) $$\theta ={tan}^{-1}\frac{1}{f}$$

When the size of the imaging element is the same, the focal length and angle of view are inversely proportional, as shown in Equation (2). As the focal length increased, the angle of view narrowed [25]. As the focal length decreased, the angle of view increased [26]. In a zoomed-in optical system with an infinite focal length, the end with the shortest focal length is called the wide end [27]. The end with the longest focal length is called the telephoto end [28]. The region between the wide and telephoto ends is called the intermediate end (normal or middle end) [29].

**Figure 1:**
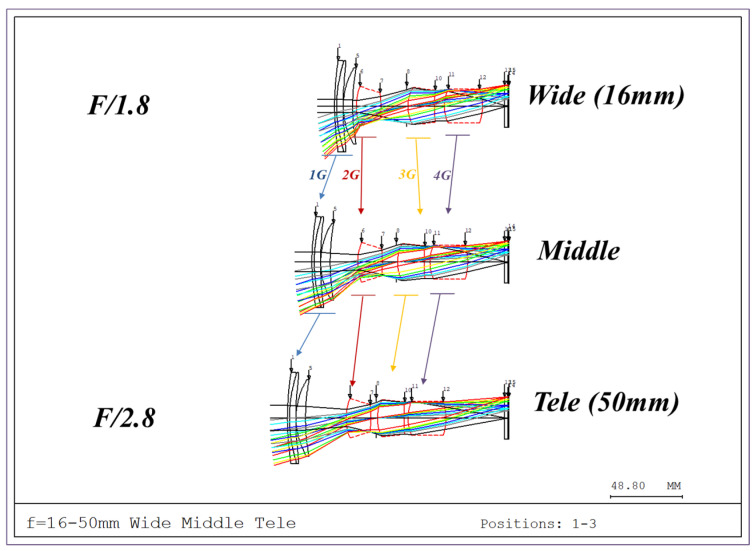
Optical layout of our designed optical system.

Figure 1 shows the optical path of the designed system. The zoom lens comprises a four-group configuration. From the object side to the image side, the configuration includes a first lens group (G1) with positive power, a second group (G2) with negative power, a third group (G3) with positive power, and a fourth group (G4) with positive power. As the spacing between lens groups changes during zooming, the fourth group moves toward the object, and the focal length changes from wide-angle to telephoto.

When designing a zoom lens, it is essential to maintain consistent performance throughout the zoom range, not just at the wide and telephoto ends. Therefore, the zoom locus—the spacing between lens groups—must be calculated to allow continuous focal length adjustment. Regions where performance degradation may occur must be identified, and zoom adjustments applied accordingly to improve optical performance throughout the zoom process.

Camera systems must be capable of capturing objects at relatively close ranges. Changes in the object distance can cause defocus on the image plane; therefore, defocus may occur due to variations in the distance between the object and the camera [30]. To compensate for this, the position of the image plane can be fixed by moving a specific lens group, designated as the focusing group [5].

**Figure 2:**
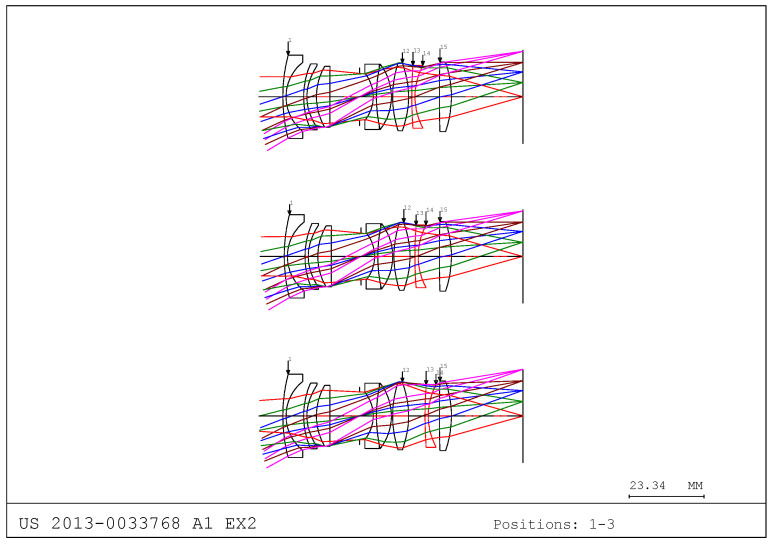
Focusing group movement according to object distance change.

In this study, we referred to the optical layout from US Patent 2013-0033768 A1 EX2 [31]. Accordingly, we present an example of an inner-focusing structure using this optical path. As shown in Figure 2, when the object distance decreases, the 11th surface moves toward the image plane to compensate for the shift in the focal plane. This approach represents an inner-focusing method, in which the internal lens group moves to achieve focus without altering the overall length of the optical system. This contrasts with front-focusing methods, which move the first lens group, or rear-focusing methods, which move the last lens group. The inner-focusing structure is advantageous in terms of environmental durability and mechanical stability.

**Figure 3:**
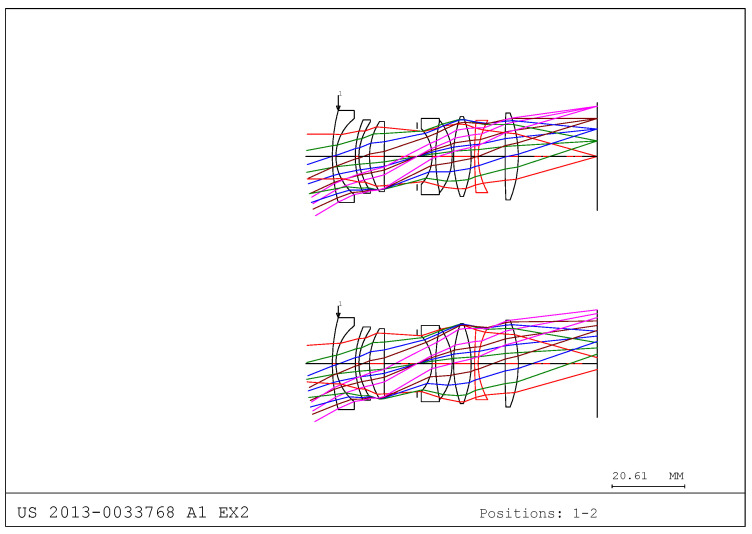
Optical layout is changed when there is no moving group due to object distance change.

However, as shown in Figure 3, if there is no moving lens group despite the change in the object distance, the image plane moves, making accurate focusing impossible. Therefore, the movement of specific lens groups is essential to compensate for the defocusing of the image plane as the object distance changes.

**Figure 4:**
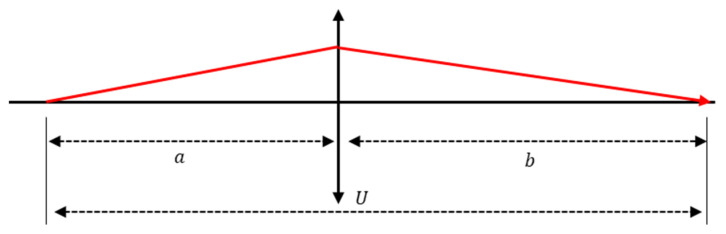
Refraction of light in an arbitrary thin lens.

The selection of the focusing groups was based on field sensitivity and aberration sensitivity [32]. A group of lenses that do not change significantly in aberration depending on movement and mainly affect only the back focal length (BFL) is suitable as a focusing group. If the positions of the viewpoint and imaging elements are different, the image becomes blurred, and the resolution is reduced accordingly. Therefore, focal sensitivity and the ratio of the movement of a specific lens group to the movement of the image plane must be calculated when determining the focusing group. Figure 4 shows the optical path for an arbitrary lens group, where *a* is the object distance from the image formed by the preceding lens groups, and *b* is the image distance to the image formed by an arbitrary lens.

In this case, *U* is the total distance from the object to the surface and can be expressed as follows:

(3) U≡a+b

If the sign convention is based on the starting point of the light ray and the focal length of an arbitrary group is *f*, the imaging equation can be expressed as follows, using the object distance *a* and the image distance *b*.

(4) $$\frac{1}{a}+\frac{1}{b}=\frac{1}{f}$$

The transverse magnification m of an optical system is defined as the image distance *b* divided by the object distance *a*, as expressed in Equation (5). If Equation (4) is differentiated based on the object distance *a* and image distance *b*, we obtain Equation (6). The displacement of the upper surface ∆*b* relative to the movement of the object surface can be expressed in Equation (7).

(5) $$m\equiv -\frac{b}{a}$$

(6) $$-\frac{∆a}{a^{2}}+\frac{∆b}{b}=0$$

(7) $$∆b=-{\left(\frac{b}{a}\right)}^{2}∆a=-m^{2}∆a$$

Here, if this arbitrary group moves slightly in the direction of the optical axis, the change in the distance to the object, ∆*U*, can be expressed in Equation (7). Therefore, the movement of the image surface relative to that of the lens can be observed.

(8) $$∆U=∆a+∆b=∆a(1-m^{2})$$

**Figure 5:**
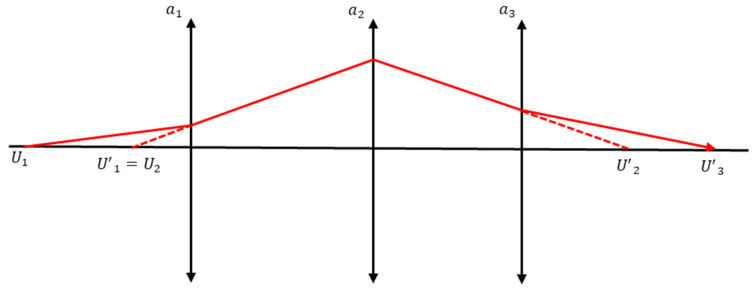
Optical path showing refraction of light for any number of lenses.

The above formula was used to calculate the magnitude of lens movement on the image plane when there was one lens group. However, if there are multiple groups in this optical system, the amount of image movement required for the movement of an arbitrary lens group must be calculated. Therefore, the calculations can be performed as follows; Figure 5 shows the optical layout of an optical system comprising the three lens groups.

Here, when the movement amount of the first group is ∆*a*_1_, the change in the material distance of the first group ∆*U*′_1_ can be expressed as Equation (9).

(9) $$∆{U^{\prime }}_{1}=(1-{m_{1}}^{2})∆a_{1}$$

However, if there are three groups, the change in the material distance to the third group due to the movement of the first group, ∆*U*′_3_ can be expressed as Equation (10).

(10) $$∆{U^{\prime }}_{3}=(1-{m_{1}}^{2}){m_{2}}^{2}{m_{3}}^{2}∆a_{1}$$

We can generalize this fact when the *i*th group in an optical system consisting of a total of *N* groups moves by ∆*a_i_*. Therefore, the amount of change in the image surface ∆*U′_N_* can be expressed as Equation (11). In Equation (12), *p_i_* is called the punt sensitivity or species sensitivity of the *i*th group [33].

(11) $$∆{U^{\prime }}_{N}=p_{i}∆a_{i}$$

(12) $$p_{i}\equiv (1-{m_{i}}^{2})\prod_{k=i+1}^{N}{m_{k}}^{2}$$

The transverse magnification of each group can be determined using a Lagrange invariant, which is a physical quantity whose value does not change before or after refraction. The Lagrange invariant is expressed by (13) and (14) [34]:

(13) $$nuy=n^{\prime }u^{\prime }y^{\prime }$$

(14) $$m=\frac{y^{\prime }}{y}=\frac{nu}{n^{\prime }u^{\prime }}$$

Therefore, the transverse magnification can be obtained by multiplying the refractive index *n* before refraction by the angle *u* of the on-axis ray divided by the product of the refractive index *n*′ after refraction and the angle *u*′ of the on-axis ray after refraction.

Using the formula described above, the focus sensitivity of each lens group (Groups 1, 2, 3, and 4) in the optical system was calculated, and the results are presented in Table 2. This refers to the change in the BFL that occurs when the group is moved by 1 mm.

This design adopts an inner-focusing method that allows focus adjustment while maintaining the overall length of the lens. Accordingly, selecting the appropriate lens group for AF was a key design consideration. The 1st and 4th groups were not suitable as AF groups because their total length changes during operation.

Both Groups 2 and 3 showed similar performance in terms of focusing sensitivity. However, because most real-world drive mechanisms use lightweight systems such as small stepping motors (STMs), the mass and diameter of the lens group directly impact driving stability. Group 3 had a relatively large diameter and heavier mass, leading to significant mechanical constraints. In contrast, Group 2 had a smaller and lighter structure, making it highly compatible with compact drive systems.

In particular, reducing the mass of the focusing group improves AF response speed, facilitates system miniaturization, and reduces operational noise due to lower motor load. In this study, Group 2 was selected as the AF driving lens group as the optimal choice that satisfies both optical performance and mechanical efficiency. This decision enabled the design of a high-performance lens suitable for lightweight mirrorless systems, successfully achieving a balance between optical quality and driving efficiency.

## 3. Results

In this study, we designed a high-speed zoom optical system with a focal length range of 16–50 mm and a bright aperture value of F/1.8–2.8 for an APS-C standard image sensor. The system is intended to deliver a brighter image than standard zoom lenses with constant apertures of F/2.0–2.8 or F/2.8, while meeting performance requirements for aberration suppression and resolution maintenance.

In this section, the optical performance of the designed system is evaluated through key performance indicators, including MTF, spherical aberration, longitudinal chromatic aberration, and distortion across the full zoom range, based on simulations performed using optical design software (CODE V) [35].

**Figure 6:**
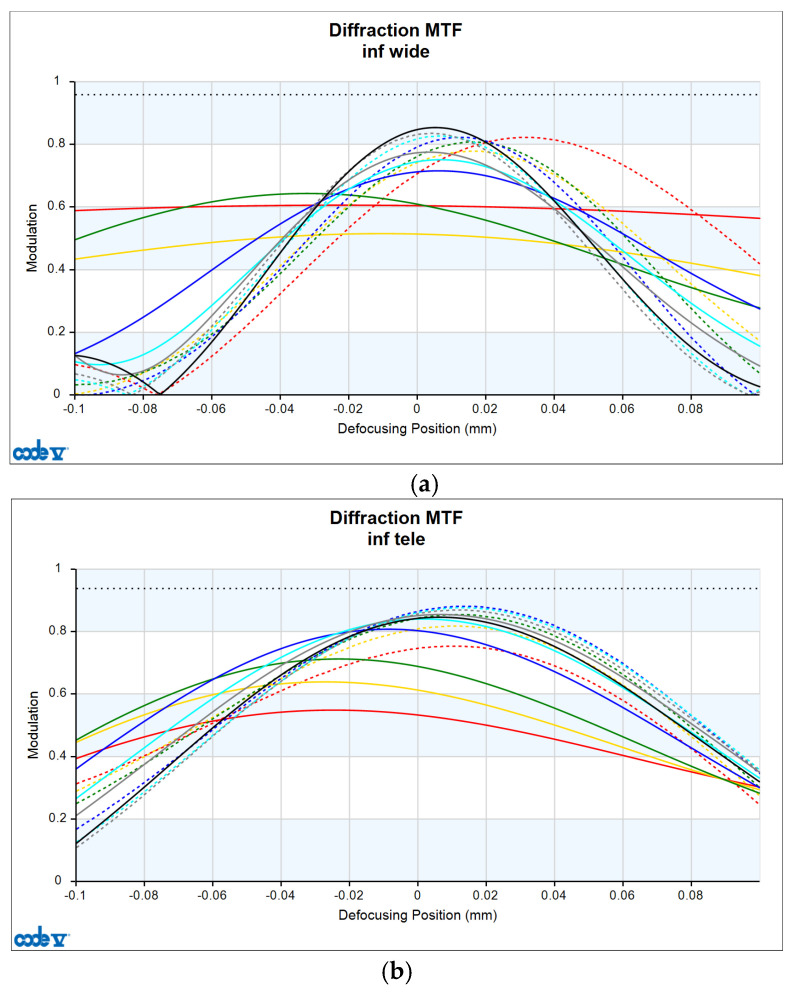
Through-focus MTF (30 lp/mm) at (**a**) wide and (**b**) telephoto ends.

The resolution of the designed optical system was assessed via MTF analysis under conditions of 10 lp/mm (representing low-frequency contrast performance) and 30 lp/mm (representing high-frequency resolution), in accordance with general optical performance evaluation criteria. Figure 6a,b show the through-focus MTF results at 30 lm/mm for the wide-angle and telephoto ends, respectively.

Under wide-angle conditions (at infinity focus), the MTF maintained a performance above 80% at 30 lp/mm in the center and above 50% across most of the periphery. Thus, stable resolution is achieved not only in the center but also at the edges, even with a bright aperture of F/1.8. In the telephoto range, the MTF performance was even higher, with a center resolution of approximately 85% at 30 lp/mm. This improvement is attributed to enhanced aberration suppression as the aperture narrows to F/2.8.

Moreover, the smooth aberration transitions across the field and the minimal difference in defocus positions between the center and periphery indicate well-controlled field curvature. Overall, the system maintained MTF performance above 50% at 30 lp/mm throughout the full zoom range, demonstrating effective aberration control suitable for high-resolution imaging. Notably, by adopting the second lens group—characterized by low aberration sensitivity—as the AF driving group, aberration changes were stably controlled even during focusing, contributing to consistent MTF performance.

**Figure 7:**
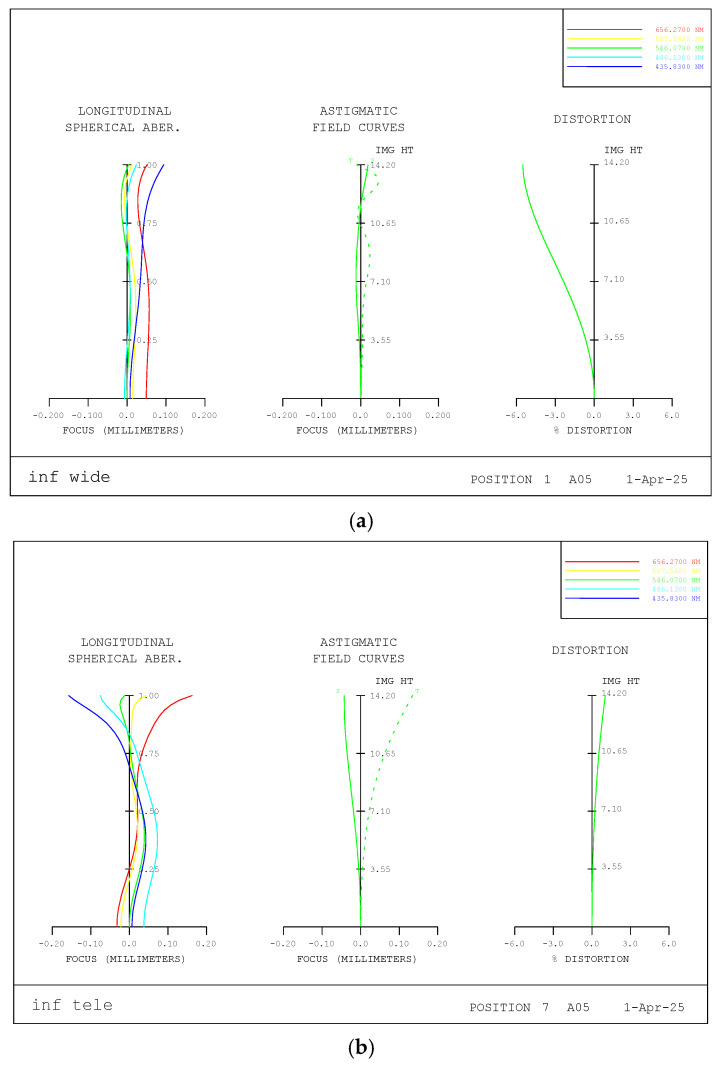
Longitudinal aberration plots at infinity point: (**a**) wide angle and (**b**) telephoto ends.

Through optical performance analysis, the aberration correction status and resolution over the entire zoom range were evaluated and compared with those of previously developed optical systems. Figure 7a,b show the longitudinal aberrations at the wide-angle and telephoto ends of the infinite point, respectively.

Looking at the aberration diagrams above, we can observe that spherical aberration and field curvature are generally well-corrected at wide angles. Therefore, image quality in both the center and periphery is adequate. At the telephoto end, spherical aberration tends to slightly advance the focus position at longer wavelengths (656.27 nm), indicating that aberration control is well maintained across other wavelengths.

Chromatic aberration can be evaluated by examining the difference in focus position near the optical axis (at an incidence height of 0.00) across different wavelengths. It is maintained within 65 μm at both the wide-angle and telephoto ends.

In this optical system, barrel-type distortion is –5.5% at the wide-angle end, which is relatively large compared to the telephoto range. This increase is attributed to the wide field-of-view characteristics of wide-angle lenses and the nonlinear magnification effects near the image periphery. Negative distortion, in general, poses less visual discomfort compared to positive distortion during image capture. In our assessment, this level of distortion can be sufficiently corrected using in-camera digital processing. Although the telephoto end shows some pincushion-type distortion, it remains well-controlled within approximately 1%.

Table 3 shows the MTF versus field at 10 lp/mm and 30 lp/mm for our design compared to currently available models. At the wide-angle end, our design has a maximum aperture of F/1.8, offering a brighter aperture than existing models. Even in the section where performance is lowest, peripheral resolution remains above 50%, without significant degradation.

Compared to the second optical system with F/2.0–2.8, the telephoto part shows slightly lower performance in the periphery but shows higher performance in the telephoto part despite being a brighter optical system. Compared to the third optical system, both the wide-angle and telephoto are F/2.8; therefore, we can observe that the performance is slightly lower in the wide-angle and telephoto sections; however, considering that F/# has been lowered, it shows sufficiently good performance.

The optical system (F/1.8–2.8) designed in this study was evaluated by comparing its performance with that of a conventional F/2.0–2.8 variable aperture lens and an F/2.8 fixed aperture lens. All comparison targets shared the same focal length range (16–50 mm), and MTF performance was analyzed at spatial frequencies of 10 lp/mm and 30 lp/mm under both wide-angle and telephoto conditions. Under wide-angle conditions, this design exhibits overall stable high-frequency response characteristics, maintaining a center MTF of over 80% and a periphery resolution of over 50%, even at the F/1.8 wide aperture. In particular, compared to the F/2.0–2.8 variable aperture design, MTF performance is maintained better both in the center and the periphery throughout the wide-angle and telephoto ranges. The F/2.8 constant aperture design has the best center performance; however, the design in this study is superior in peripheral performance in the wide-angle range.

The design is competitive in that we could achieve a similar level of resolution even under relatively brighter aperture conditions. Under telephoto conditions, the system maintains approximately 85% center MTF at F/2.8, demonstrating stable resolution characteristics across the entire field. The F/2.8 fixed aperture design shows excellent MTF from center to periphery without performance degradation; however, it is relatively limited in terms of aperture brightness compared to our design.

The proposed design achieves equivalent or better resolution performance at brighter apertures than currently developed lenses—without increasing the number of lenses or aspherical elements. Therefore, this study demonstrates both the optical efficiency and the practical feasibility of a high-speed zoom lens design strategy.

## 4. Conclusions

In this study, we designed a high-speed standard wide-angle zoom lens with a focal length of 16–50 mm and an aperture of F/1.8–2.8 for an APS-C standard image sensor. In particular, this study is the first to implement a bright aperture value of F/1.8 in the wide-angle range and is evaluated as a demonstration of overcoming the limitations of currently developed high-speed zoom lens technologies.

We focused on obtaining high resolution and aberration suppression while minimizing the total number of lenses and aspherical surfaces. By appropriately placing three aspherical lenses and one ED lens, major aberrations such as spherical aberration, chromatic aberration, field curvature, and distortion were effectively controlled throughout the entire zoom range. The MTF analysis results show that high-resolution characteristics are maintained not only at the center but also at the periphery across the wide-angle and telephoto ranges. Therefore, we suggest that consistent image quality can be expected, even in practical imaging environments.

In addition, by adopting a second lens group—insensitive to aberration changes during focus adjustment—as the AF drive group, we achieved mechanical-optical integration that simultaneously considers system stability, miniaturization, and low noise. As a result, overall optical performance remains stable during focusing, which is a significant advantage for real-world applications such as video recording or continuous shooting. Our design also provides a brighter and wider aperture while maintaining resolution equal to or better than that of current commercial products featuring a variable aperture of F/2.0–2.8 or a constant aperture of F/2.8. Especially in the wide-angle range, the lens exhibits excellent resolution at both the center and periphery, even with the aperture fully open—ensuring high image quality in low-light conditions.

In summary, this study successfully developed a high-speed zoom lens that balances the often conflicting requirements of high resolution, large aperture, lightweight construction, aberration correction, and mechanical efficiency. This design is technically significant, as it demonstrates the feasibility of achieving excellent aberration suppression and resolution while maintaining an open aperture of F/1.8 in the wide-angle range—an area where existing technologies fall short.

The results of this study can serve as a practical and feasible design reference for the development of standard zoom lenses in future high-performance mirrorless systems. Therefore, the proposed lens design represents a competitive solution for various imaging and video applications that demand the combined advantages of portability, resolution, and low-light performance in crop-sensor systems.

## Figures and Tables

**Table 1:** Comparison of key specifications and optical system configurations of standard wide-angle zoom lens design with (**a**) our designed optical system, (**b**) [13], and (**c**) [14].

Specifications	(a)	(b)	(c)
Focal length	16 mm to 50 mm	16 mm to 50 mm	16 mm to 50 mm
F/#	F/1.8–2.8	F/2.0–2.8	F/2.8
Sensor	APS-C	APS-C	APS-C
Group-element	13 groups and 17 lenses	13 groups and 18 lenses	13 groups and 16 lenses
Aspherical	3 lenses	3 lenses	4 lenses
Minimum object distance (MOD)	0.3 m	0.3 m	0.3 m
Max ratio	0.2×	0.19×	0.24×
Diameter × length	Φ61 mm × L 119. 3 mm	*Φ*81 mm × *L* 96.5 mm	*Φ*84 mm × *L* 117 mm
Optical layout	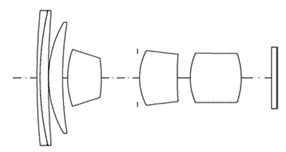	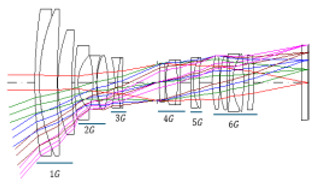	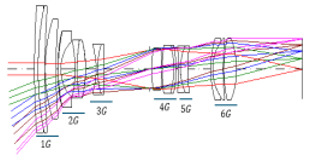

**Table 2:** Calculated results of focus sensitivity of designed optical system.

Lens Group	Wide	Normal	Tele
G1	0.02361	0.07046	0.20411
G2	0.86926	1.65251	3.69190
G3	–0.89014	–1.60840	1.65251
G4	0.997266	0.88542	0.30165

**Table 3:** MTF vs. field performance comparisons under wide-angle and telephoto conditions with (**a**) our design, (**b**) [13], and (**c**) [14].

	(a)	(b)	(c)
Wide	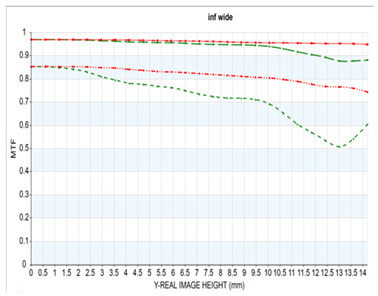	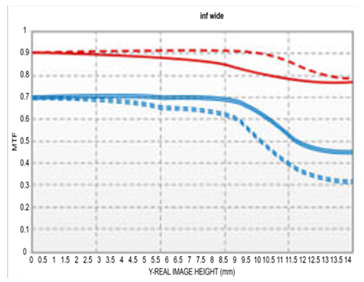	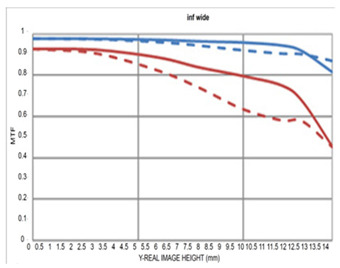
Tele	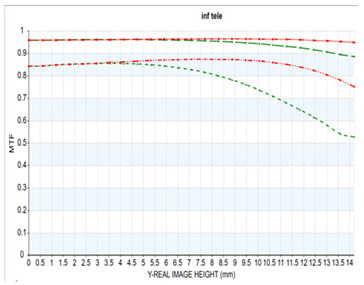	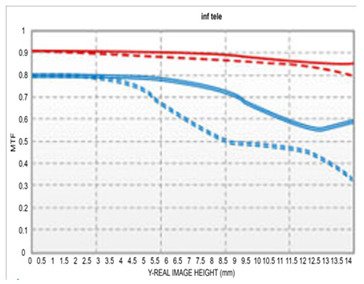	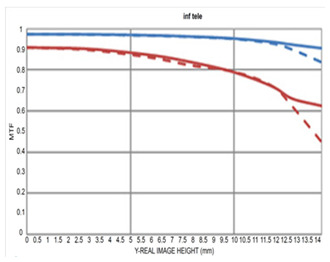

## Data Availability

The data presented in this study are included in the article.

## Abbreviations

The following abbreviations are used in this manuscript:

MTF	Modulation transfer function
F/#	f-number
ED	Extra-low dispersion
APS-C	Advanced photo system type-C
MOD	Minimum object distance
BFL	Back focal length
EFL	Effective focal length
AF	Autofocus
STM	Stepping motor
